# Pneumonia detection in chest X-ray images using an ensemble of deep learning models

**DOI:** 10.1371/journal.pone.0256630

**Published:** 2021-09-07

**Authors:** Rohit Kundu, Ritacheta Das, Zong Woo Geem, Gi-Tae Han, Ram Sarkar

**Affiliations:** 1 Department of Electrical Engineering, Jadavpur University, Kolkata, India; 2 Department of Computer Science & Engineering, Jadavpur University, Kolkata, India; 3 College of IT Convergence, Gachon University, Seongnam, South Korea; Politechnika Slaska, POLAND

## Abstract

Pneumonia is a respiratory infection caused by bacteria or viruses; it affects many individuals, especially in developing and underdeveloped nations, where high levels of pollution, unhygienic living conditions, and overcrowding are relatively common, together with inadequate medical infrastructure. Pneumonia causes pleural effusion, a condition in which fluids fill the lung, causing respiratory difficulty. Early diagnosis of pneumonia is crucial to ensure curative treatment and increase survival rates. Chest X-ray imaging is the most frequently used method for diagnosing pneumonia. However, the examination of chest X-rays is a challenging task and is prone to subjective variability. In this study, we developed a computer-aided diagnosis system for automatic pneumonia detection using chest X-ray images. We employed deep transfer learning to handle the scarcity of available data and designed an ensemble of three convolutional neural network models: GoogLeNet, ResNet-18, and DenseNet-121. A weighted average ensemble technique was adopted, wherein the weights assigned to the base learners were determined using a novel approach. The scores of four standard evaluation metrics, precision, recall, f1-score, and the area under the curve, are fused to form the weight vector, which in studies in the literature was frequently set experimentally, a method that is prone to error. The proposed approach was evaluated on two publicly available pneumonia X-ray datasets, provided by Kermany et al. and the Radiological Society of North America (RSNA), respectively, using a five-fold cross-validation scheme. The proposed method achieved accuracy rates of 98.81% and 86.85% and sensitivity rates of 98.80% and 87.02% on the Kermany and RSNA datasets, respectively. The results were superior to those of state-of-the-art methods and our method performed better than the widely used ensemble techniques. Statistical analyses on the datasets using McNemar’s and ANOVA tests showed the robustness of the approach. The codes for the proposed work are available at https://github.com/Rohit-Kundu/Ensemble-Pneumonia-Detection.

## Introduction

**Fig 1:**
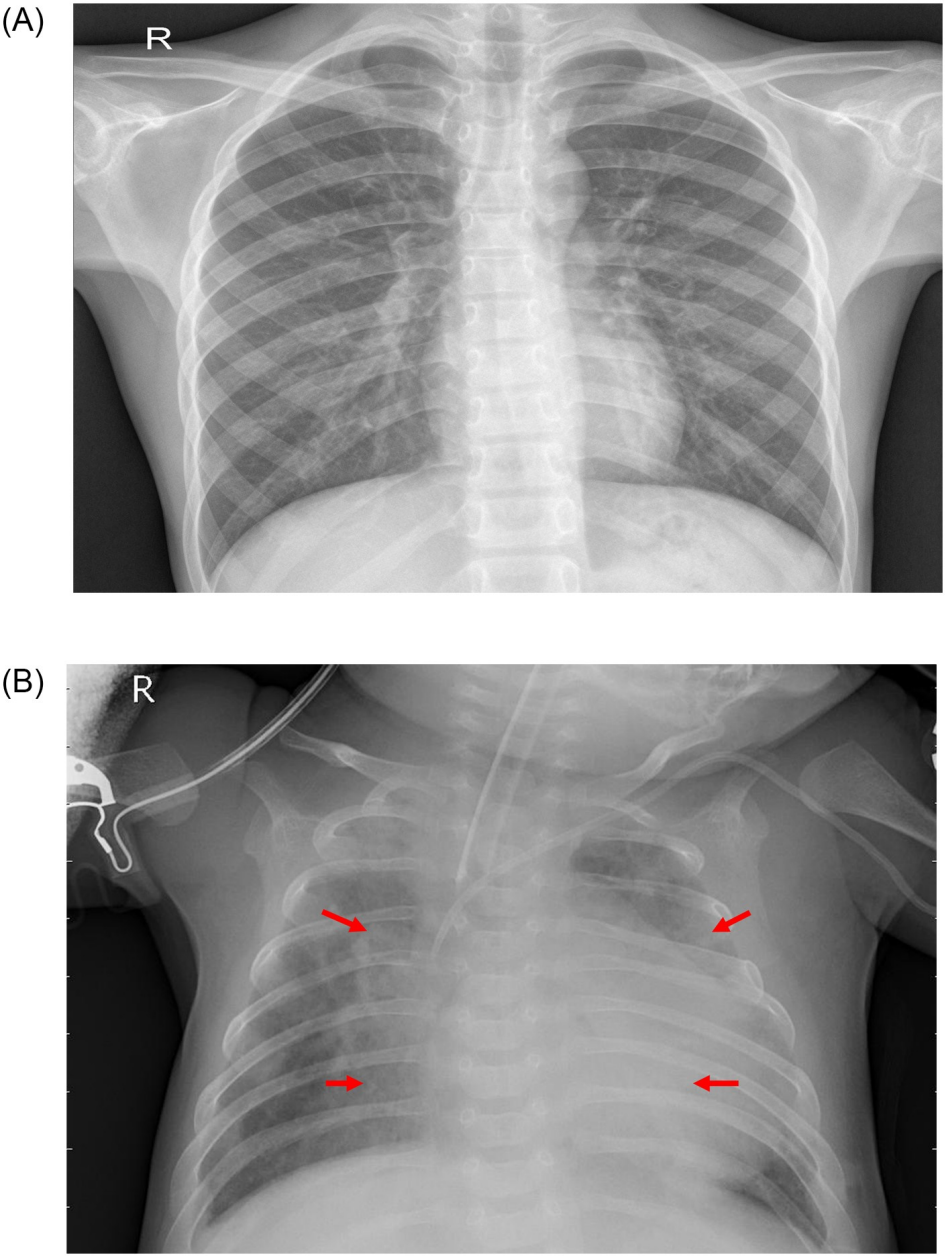
Examples of two X-ray plates that display (a) a healthy lung and (b) a pneumonic lung. The red arrows in (b) indicate white infiltrates, a distinguishing feature of pneumonia. The images were taken from the Kermany dataset [4].

Pneumonia is an acute pulmonary infection that can be caused by bacteria, viruses, or fungi and infects the lungs, causing inflammation of the air sacs and pleural effusion, a condition in which the lung is filled with fluid. It accounts for more than 15% of deaths in children under the age of five years [1]. Pneumonia is most common in underdeveloped and developing countries, where overpopulation, pollution, and unhygienic environmental conditions exacerbate the situation, and medical resources are scanty. Therefore, early diagnosis and management can play a pivotal role in preventing the disease from becoming fatal. Radiological examination of the lungs using computed tomography (CT), magnetic resonance imaging (MRI), or radiography (X-rays) is frequently used for diagnosis. X-ray imaging constitutes a non-invasive and relatively inexpensive examination of the lungs. Fig 1 shows an example shows an example of a pneumonic and a healthy lung X-ray. The white spots in the pneumonic X-ray (indicated with red arrows), called infiltrates, distinguish a pneumonic from a healthy condition. However, chest X-ray examinations for pneumonia detection are prone to subjective variability [2, 3]. Thus, an automated system for the detection of pneumonia is required. In this study, we developed a computer-aided diagnosis (CAD) system that uses an ensemble of deep transfer learning models for the accurate classification of chest X-ray images.

Deep learning is an important artificial intelligence tool, which plays a crucial role in solving many complex computer vision problems [5, 6]. Deep learning models, specifically convolutional neural networks (CNNs), are used extensively for various image classification problems. However, such models perform optimally only when they are provided with a large amount of data. For biomedical image classification problems, such a vast amount of labeled data is difficult to acquire because it requires that expert doctors classify each image, which is an expensive and time-consuming task. Transfer learning is a work-around to surmount this obstacle. In this technique, to solve a problem that involves a small dataset, a model trained on a large dataset is re-used and the network weights determined in this model are applied. CNN models trained on a large dataset such as ImageNet [7], which consists of more than 14 million images, are frequently used for biomedical image classification tasks.

**Fig 2:**
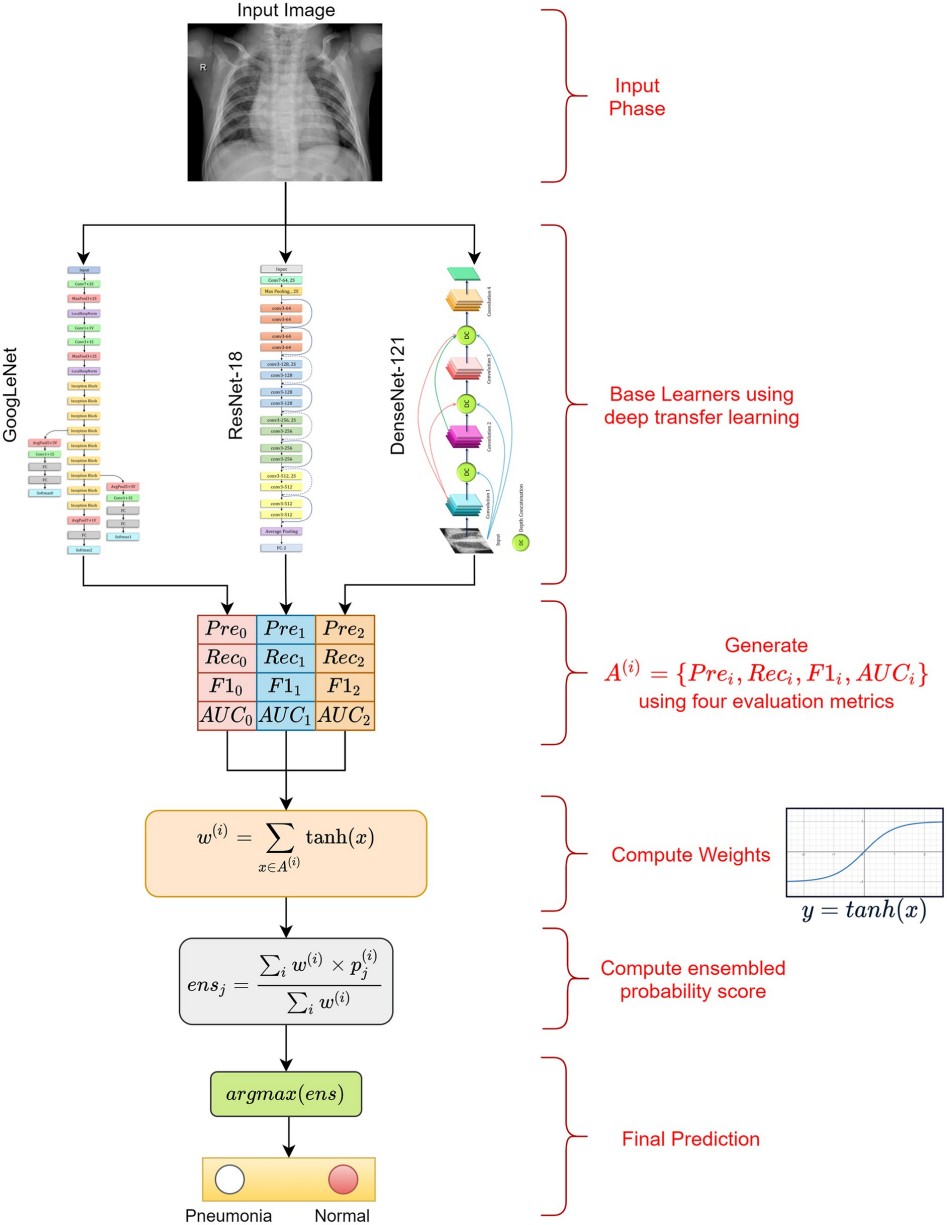
Representation of the proposed pneumonia detection framework. *Pre* = Precision score, *Rec* = Recall score, *F*1 = F1-score, *AUC* = AUC score, and *A*^(*i*)^ = {*Pre*_*i*_, *Rec*_*i*_, *F*1_*i*_, *AUC*_*i*_}; *w*^(*i*)^ is the weight generated for the *i*^*th*^ base learner to compute the ensemble, $p_{j}^{\left(i\right)}$ is the probability score for the *j*^*th*^ sample by the *i*^*th*^ classifier, and *ens*_*j*_ is the fused probability score for the *j*^*th*^ sample; and the *argmax* function returns the position having the highest value in a 1D array, i.e., in this case it generates the predicted class of the sample.

Ensemble learning is a popular strategy in which the decisions of multiple classifiers are fused to obtain the final prediction for a test sample. It is performed to capture the discriminative information from all the base classifiers, and thus, results in more accurate predictions. Some of the ensemble techniques that were most frequently used in studies in the literature are average probability, weighted average probability, and majority voting. The average probability-based ensemble assigns equal priority to each constituent base learner. However, for a particular problem, a certain base classifier may be able to capture information better than others. Thus, a more effective strategy is to assign weights to all the base classifiers. However, for ensuring the enhanced performance of the ensemble, the value of the weights assigned to each classifier is the most essential factor. Most approaches set this value based on experimental results. In this study, we devised a novel strategy for weight allocation, where four evaluation metrics, precision, recall, f1-score, and area under receiver operating characteristics (ROC) curve (AUC), were used to assign the optimal weight to three base CNN models, GoogLeNet, ResNet-18, and DenseNet-121. In studies in the literature, in general, only the classification accuracy was considered for assigning weights to the base learners [8], which may be an inadequate measure, in particular when the datasets are class-imbalanced. Other metrics may provide better information for prioritizing the base learners. The overall workflow of the proposed ensemble framework is presented in Fig 2.

## Related work

Pneumonia detection using chest X-rays has been an open problem for many years [9, 15], the main limitation being the scarcity of publicly available data. Traditional machine learning methods have been explored extensively. Chandra et al. [16] segmented the lung regions from chest X-ray images and extracted eight statistical characteristics from these regions, which they used to classify them. They implemented five traditional classifiers: multi-layer perceptron (MLP), random forest, sequential minimal optimization (SMO), classification via regression, and logistic regression. They evaluated their method on 412 images and achieved a 95.39% accuracy rate using the MLP classifier. Kuo et al. [17] used 11 features to detect pneumonia in 185 schizophrenia patients. They applied these features in a large number of regression and classification models, such as decision trees, support vector machines, and logistic regression, and compared the results of the models. They achieved the highest accuracy rate, 94.5%, using a decision tree classifier; the other models fell short by large margins. Similarly, Yue et al. [18] used 6 features to detect pneumonia in chest CT scan images of 52 patients; the best AUC value they achieved was 97%. However, these methods cannot be generalized and were evaluated on small datasets.

In contrast to machine learning algorithms, for which handcrafted features need to be extracted and selected for classification or segmentation [27, 28], deep learning-based methods perform end-to-end classification [29, 30], where the relevant and informative features are automatically extracted from the input data and classified. CNNs are preferred for image data classification because they automatically extract translationally invariant features through the convolution of the input image and filters. CNNs are translationally invariant and perform better than machine learning or traditional image processing methods in image classification tasks and thus are widely used by researchers.

Sharma et al. [19] and Stephen et al. [20] devised simple CNN architectures for the classification of pneumonic chest X-ray images. They used data augmentation to compensate for the scarcity of data. Sharma et al. obtained a 90.68% and Stephen et al. a 93.73% accuracy rate on the dataset provided by Kermany et al. [4], hereafter called the Kermany dataset. Data augmentation, however, provides only a limited amount of new information from which the CNNs can learn and thus may not significantly boost their performance. Rajpukar et al. [14] used the DenseNet-121 CNN model for pneumonia classification but achieved only a 76.8% f1-score for classification. They suspected that the unavailability of patient history was a major cause for the inferior performance of both their deep learning model and the radiologists with which they compared the performance of their method.

Janizek et al. [21] proposed a framework based on adversarial optimization to remove the dependency of models on the source of the datasets and produce robust predictions. They obtained a 74.7% AUC score in the source domain and a 73.9% AUC score in the target domain. Zhang et al. [22] proposed a confidence-aware module for anomaly detection in lung X-ray images, posing the detection task as a one-class problem (determining only the anomalies). They achieved an 83.61% AUC score on their dataset. Tuncer et al. [23] used a machine learning-based method in which they applied the fuzzy tree transformation to the images, followed by an exemplar division. Then, they extracted features using a multikernel local binary pattern and classified the samples using traditional classifiers. They evaluated the method on a small dataset consisting of COVID-19 and pneumonia samples and showed that it achieved a 97.01% accuracy rate.

To solve the data scarcity problem in biomedical image classification tasks, transfer learning, wherein knowledge gained from a large dataset is used to fine-tune the model on a current small dataset, is currently a frequently used approach. Recently, Rahman et al. [10], Liang et al. [11], Ibrahim et al. [12], and Zubair et al. [13] applied purely transfer learning approaches in which different CNN models pre-trained on ImageNet [7] data are used for pneumonia classification. Table 1 tabulates the development of the state of the art for the pneumonia detection problem.

**Table 1:** Existing methods for pneumonia detection.

Method	Approach	Merits	Demerits
Albahli et al. [9]	• Transfer Learning using InceptionResNet-V2	Reuse of models pretrained on a large dataset	Oversimplified for a complex pattern recognition task; Performance obtained is poor and not fit for practical use
Rahman et al. [10]	• Transfer Learning using DenseNet-201		
Liang et al. [11]	• Transfer learning using ResNet-50 pre-trained on ChestX-ray14 dataset		
Ibrahim et al. [12]	• Transfer learning using AlexNet		
Zubair et al. [13]	• Transfer learning using VGG-16		
Rajpukar et al. [14]	• Transfer learning using DenseNet-121		
Albahli et al. [15]	• Used generative adversarial networks to generate synthetic data. • Classification using ResNet-152	Generation of synthetic data to balance the classes of the data because medical data are scarce	Classification results (41% accuracy rate) are not fit for practical use
Chandra et al. [16]	• Segmentation of lung X-rays using image processing • Extraction and classification of eight statistical features	Segmentation of lungs before classification allows localization of the disease	The use of handcrafted features limits its ability to perform in complex pattern recognition tasks; Evaluation on a small dataset (412 images) cannot be generalized
Kuo et al. [17]	• Used 11 features from patient data to fit traditional classifiers	Use of 10-fold cross validation with 3 repeats avoids overfitting	Patient data are often private and not publicly available to fit to classification models
Yue et al. [18]	• Segmented lung lobes using U-Net • Extracted and classified radiomic features from CT-scan images	Segmentation before classification helps extract important features for radiologists and allows localization of the disease	Method evaluated on a small dataset (72 lesion segments) and thus difficult to generalize
Sharma et al. [19]	• Devised a CNN model for classification of X-ray images	Automatic feature learning for complex tasks	Simple linearly progressing CNN model increases computation cost without providing strong boost to performance
Stephen et al. [20]	• Developed a simple seven-layer CNN model for classification of X-ray images		
Janizek et al. [21]	• Developed a deep learning framework based on adversarial optimization	Adversarial optimization removed dependency on the source of the dataset and view of the X-rays for classification	Results (AUC 74.7%) are not fit for deployment in the field
Zhang et al. [22]	• Developed a confidence-aware module for anomaly detection in lung X-ray images	Posing the detection task as a one-class problem helped improve the model performance	The sensitivity obtained on the dataset was too low (71.70%) for practical use
Tuncer et al. [23]	• Applied fuzzy tree transformation to X-ray images • Extracted local features for classification using an ensemble of traditional classifiers	Generation of three different feature images improves the model performance	Handcrafted feature extraction limits performance in complex pattern recognition tasks; Evaluation on a small dataset cannot be generalized
Jaiswal et al. [24]	• Developed a mask region-based CNN for segmentation • Used an ensemble model for image thresholding	Use of threshold value in background boosts the performance	An irregular trend was observed, where results of the training set were lower than those of the testing set
Gabruseva et al. [25]	• Localized pulmonary opacity based on a single-shot detector • Used a snapshot ensemble model for segmentation	One-shot detector alleviates the problem of scarcity of data	Irregular trend of validation loss over epochs during model training
Pan et al. [26]	• Used an ensemble of Inception-ResNet v2, XceptionNet, and DenseNet-169 for bounding box prediction	Ensemble learning allows the fusion of salient properties of all its base learners	Pan et al. [26] suspect that their model evaluated on only one dataset may not generalize over data acquired from a different source

Most state-of-the-art deep learning methods for pneumonia detection focus on the use of a single CNN model. Ensemble learning [31, 32] allows the decisions generated by multiple CNN models to be fused, thus effectively incorporating in the ensemble model the salient features of all its base models, capturing complementary information from the different classifiers, and allowing a more robust decision. This paradigm has been seldom explored in relation to the pneumonia detection task. Jaiswal et al. [24] used a mask region-based CNN for the detection of pneumonia traces via segmentation, wherein they used an ensemble model consisting of ResNet-50 and ResNet-101 for image thresholding. Gabruseva et al. [25] proposed a deep learning framework for the localization of pulmonary opacity, which was based on a single-shot detector RetinaNet with Se-ResNext101 encoders. They executed an ensemble of several checkpoints during the training phase (snapshot ensembling) and achieved a mean average precision (mAP) of 0.26 over several intersection over union thresholds, one of the best results in the Radiological Society of North America (RSNA) Pneumonia Detection Challenge. On the same challenge, Pan et al. [26] used an ensemble of the Inception-ResNet v2, XceptionNet, and DenseNet-169 models for pneumonia detection and obtained the best result in the challenge, an mAP value of 0.33. However, ensemble models have not been used for classification tasks in the pneumonia detection problem to the best of our knowledge, and, for the first time in this domain, we adopted ensemble learning in this study for the classification of lung X-rays into “Pneumonia” and “Normal” classes. Three state-of-the-art CNN models with transfer learning, GoogLeNet, ResNet-18, and DenseNet-121, were used to form the ensemble using a weighted average probability technique, in which the weights are allocated using a novel approach.

### Motivation and contributions

As previously mentioned, pneumonia affects a large number of individuals, especially children, mostly in developing and underdeveloped countries characterized by risk factors such as overcrowding, poor hygienic conditions, and malnutrition, coupled with the unavailability of appropriate medical facilities. Early diagnosis of pneumonia is crucial to cure the disease completely. Examination of X-ray scans is the most common means of diagnosis, but it depends on the interpretative ability of the radiologist and frequently is not agreed upon by the radiologists. Thus, an automatic CAD system with generalizing capability is required to diagnose the disease. To the best of our knowledge, most previous methods in the literature focused on developing a single CNN model for the classification of pneumonia cases, and the use of the ensemble learning paradigm in this classification task has not been explored. However, the ensemble learning model incorporates the discriminative information from all the constituent base learners, allowing it to make superior predictions, and thus was implemented in this study. To handle the low amount of available biomedical data, transfer learning models were used as base learners, the decision scores of which were ensembled.

The main contributions of this study are as follows.

1. An ensemble framework, proposed for boosting the performance of the base CNN learners in pneumonia classification, was developed. For this purpose, a weighted average ensemble technique was adopted.
2. The weights assigned to the classifiers were determined by fusing four evaluation metrics: precision, recall, f1-score, and AUC. Instead of setting the weights based solely on the accuracy of classifiers or according to the results of experiments, we used a hyperbolic tangent function.
3. The proposed model was evaluated on two publicly available chest X-ray datasets, the Kermany dataset [4] and the RSNA Pneumonia Detection Challenge [33] dataset, using the five-fold cross-validation setting. The results are superior to those of state-of-the-art methods, indicating the viability of the method for use in the practical field.

## Proposed method

In this study, we designed an ensemble framework of three classifiers (Fig 2), GoogLeNet [34], ResNet-18 [35], and DenseNet-121 [36], using a weighted average ensemble scheme wherein the weights allocated to the classifiers are generated using a novel scheme, as explained in detail in the following sections.

### GoogLeNet

**Fig 3:**
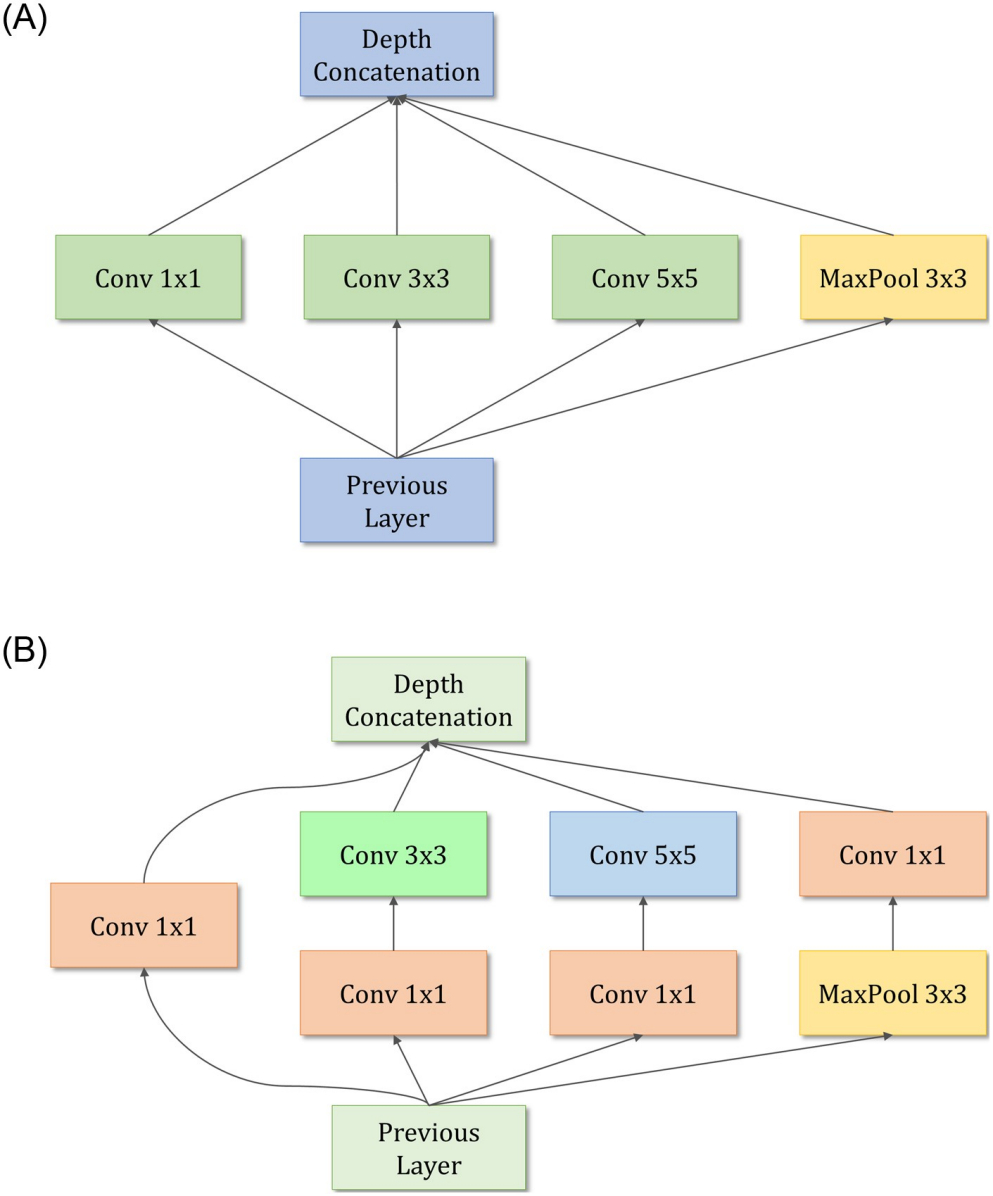
Inception modules in the GoogLeNet architecture. (a) The naive inception block that is replaced by (b) the dimension reduction inception block in the GoogLeNet architecture to improve computational efficiency.

**Fig 4:**
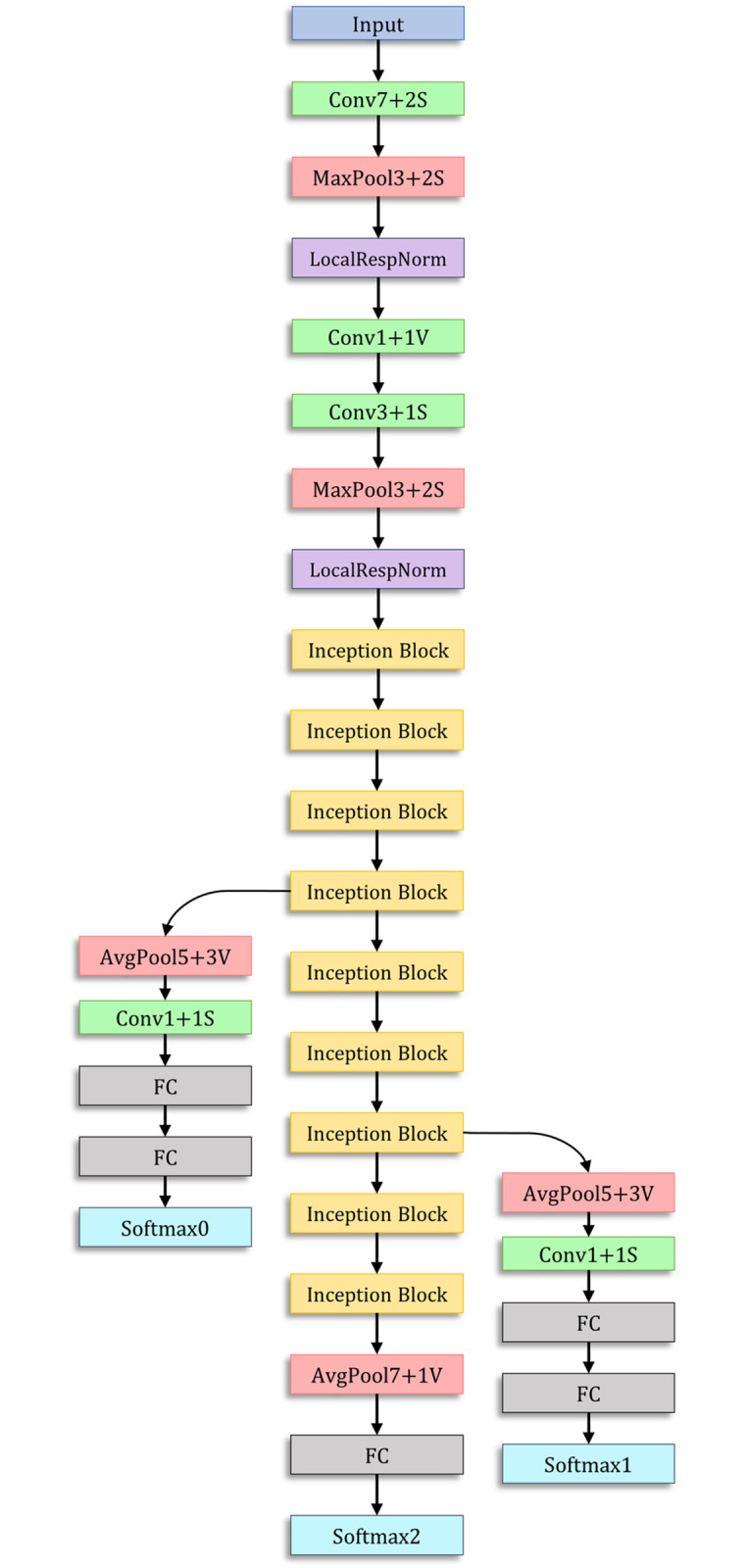
Architecture of the GoogLeNet model used in this study. The inception block is shown in Fig 3(b).

The GoogLeNet architecture proposed by Szegedy et al. [34] is a 22-layer deep network consisting of “inception modules,” instead of uniformly progressive layers. An inception block accommodates a large number of units at each stage by hosting parallel convolution and pooling layers, resulting in an uncontrolled computational complexity because of the increased number of parameters. To control the computational complexity, the GoogLeNet model uses inception blocks with dimension reduction, as shown in Fig 3(b), rather than the naive inception block (Fig 3(a)) used in [37]. The performance of GoogLeNet, in which the inception block was introduced, proves that an optimal sparse architecture built from the available dense building blocks improves the performance of artificial neural networks for computer vision tasks. The architecture of the GoogLeNet model is presented in Fig 4.

### ResNet-18

**Fig 5:**
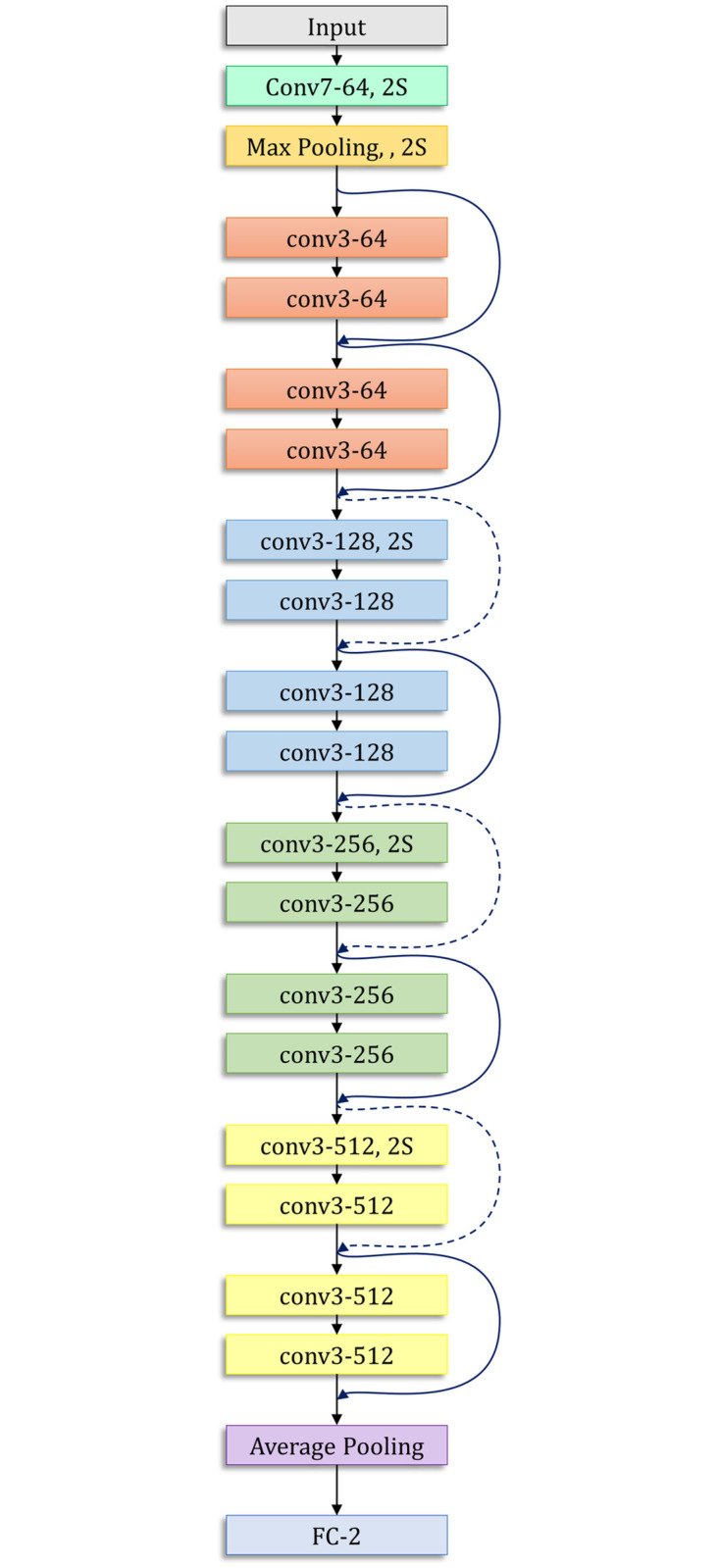
Architecture of the ResNet-18 model used in this study.

The ResNet-18 model proposed by He et al. [35] is based on a residual learning framework, which increases the efficiency of deep network training. The residual blocks in the ResNet models facilitate the optimization of the overall network, which in turn improves model accuracy, unlike the original unreferenced mapping in monotonically progressive convolutions. These residuals or “skip connections” perform identity mapping, which neither adds parameters nor increases the computational complexity. The architecture of the ResNet-18 model is presented in Fig 5.

### DenseNet-121

**Fig 6:**
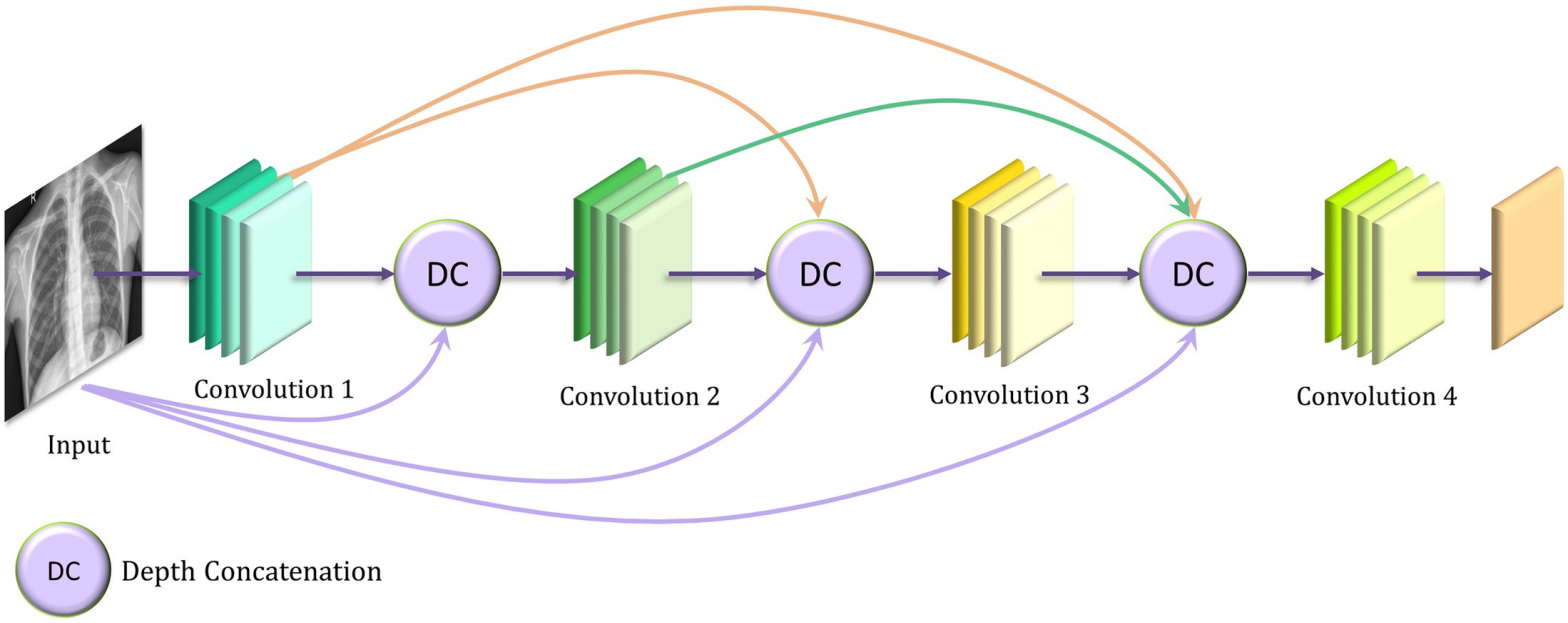
Basic architecture of the DenseNet convolutional neural network model.

The DenseNet architectures proposed by Huang et al. [36] provide a rich feature representation while being computationally efficient. The primary reason is that, in each layer of the DenseNet model, the feature maps in the current layer are concatenated with those from all the preceding layers, as shown in Fig 6. Because fewer channels are accommodated in the convolutional layers, the number of trainable parameters is diminished, and thus, the model is computationally efficient. Furthermore, the concatenation of the feature maps from the previous layers with the current layer enhances the feature representation.

The values of the hyperparameters used for training the learning algorithms (base learners) were set empirically and are shown in Table 2.

**Table 2:** Hyperparameters used for training the convolutional neural network base learners.

Hyperparameter	Value
Optimizer	Adam
Loss Function	Cross Entropy
Initial Learning Rate	0.0001
Learning Rate Scheduler	ReduceLROnPlateau
No. of Epochs	30

### Proposed ensemble scheme

The ensemble learning model helps incorporate the discriminative information of all its constituent models, and thus, its predictions are superior to those of any of its constituent base learners. Weighted average ensembling is a powerful classifier fusion mechanism. However, the choice of the weights to be allocated to the respective base learners plays a pivotal role in ensuring the success of the ensemble. Most approaches in the literature set the weights experimentally or based solely on the accuracy of the classifier. However, this may not be a good measure when a class imbalance exists in the dataset. The use of other evaluation measures, such as precision, recall (sensitivity), f1-score, and AUC, may provide relatively robust information for determining the priority of the base learners. To this end, in this study, we devised a novel strategy for weight allocation, which is explained in the following.

First, the probability scores obtained during the training phase by the base learners are utilized to calculate the weights assigned to each base learner using the proposed strategy. These generated weights are used in the formation of an ensemble trained on the test set. This strategy is implemented to ensure that the test set remains independent for predictions. The predictions of the *i*^*th*^ model (${\hat{y}}^{i}$) are generated and compared with the true labels (*y*) to generate the corresponding precision score (*pre*^(*i*)^), recall score (*rec*^(*i*)^), f1-score (*f*1^(*i*)^), and AUC score (*AUC*^(*i*)^). Assume that this forms an array *A*^(*i*)^ = {*pre*^(*i*)^, *rec*^(*i*)^, *f*1^(*i*)^, *AUC*^(*i*)^}. The weight (*w*^(*i*)^) assigned to each classifier is then computed using the hyperbolic tangent function, as shown in Eq 1. The range of the hyperbolic tangent function is [0, 0.762] because *x* represents an evaluation metric, the value of which is in the range [0, 1]. It monotonically increases in this range; thus, if the value of a metric *x* is high, the *tanh* function rewards it by assigning to it a high priority; otherwise, the function penalizes it.

$$\begin{array}{c} \begin{array}{cc} w^{\left(i\right)} & =\sum_{x\in A^{\left(i\right)}}\tanh\left(x\right)\\ & =\sum_{x\in A^{\left(i\right)}}\frac{e^{x}-e^{-x}}{e^{x}+e^{-x}} \end{array} \end{array}$$ (1)

These weights (*w*^(*i*)^) computed by Eq 1 are multiplied by the decision scores of the corresponding base learners to compute the weighted average probability ensemble, as shown in Eq 2, where the probability array (for a binary class dataset) of the *j*^*th*^ test sample by the *i*^*th*^ base classifier is $p_{j}^{\left(i\right)}=\left\{a,1-a\right\}$, where *a* ≤ 1 and the ensemble probability for the sample is *ensemble*_*prob*_*j*_ = {*b*, 1 − *b*}.

$$\begin{array}{c} ensemble\_prob_{j}=\frac{\sum_{i}w^{\left(i\right)}\times p_{j}^{\left(i\right)}}{\sum_{i}w^{\left(i\right)}} \end{array}$$ (2)

Finally, the class predicted by the ensemble is computed by Eq 3, where *prediction*_*j*_ denotes the predicted class of the sample.

$$\begin{array}{c} prediction_{j}=argmax\left(ensemble\_prob_{j}\right) \end{array}$$ (3)

## Results and discussion

In this section, we report the evaluation results of the proposed method. Two publicly available pneumonia chest X-ray datasets were used. The first dataset, the Kermany dataset [4], consists of 5856 chest X-ray images from a large population of both adults and children, unevenly distributed among the classes “Pneumonia” and “Normal.” The second dataset was provided by the RSNA [33] and was posed as a Kaggle challenge for pneumonia detection. The distribution of images in the two datasets is provided in Table 3. The description of images in the training and testing sets of each fold of the 5-fold cross-validation scheme adopted in this study are also shown in the table. Furthermore, the implications of the obtained results are discussed. A comparative evaluation was conducted to demonstrate the superiority of the proposed method over other models and frequently used ensemble techniques published in the literature.

**Table 3:** Description of images in the training and testing sets in each fold of five-fold cross-validation in the two datasets used in this study.

Dataset	Division	Class	No. of Images	Size of Images (Range)	Size of Resized Images
**Kermany**	Train	Normal	1267	(127×384×3)—(2713×2517×3)	224×224×3
		Pneumonia	3419		
	Test	Normal	316	(189×490×3)—(2458×2720×3)	224×224×3
		Pneumonia	854		
	**Total Images**		**5856**		
**RSNA**	Train	Lung Opacity	16488	(1024×1024×3)	224×224×3
		No Lung Opacity	4801		
	Test	Lung Opacity	4111	(1024×1024×3)	224×224×3
		No Lung Opacity	1201		
	**Total Images**		**26601**		

### Evaluation metrics

To evaluate the proposed ensemble method on the two pneumonia datasets, four standard evaluation metrics were used: accuracy (*Acc*), precision (*Pre*), recall (*Rec*), and f1-score (*F*1). To define these evaluation metrics, first, we define the terms “True Positive,” “False Positive,” “True Negative,” and “False Negative.”

For a binary classification task, suppose the two classes in the dataset are called the “positive” and the “negative” class. The aforementioned terms can then be defined as follows.

- *True Positive* (TP) refers to a sample belonging to the positive class, being correctly classified by a model.
- *False Positive* (FP) refers to a sample belonging to the negative class, being incorrectly classified as belonging to the positive class.
- *True Negative* (TN) refers to a sample belonging to the negative class, being correctly classified by the model.
- *False Negative* (FN) refers to a sample belonging to the positive class, being incorrectly classified as belonging to the negative class.

Now, the four evaluation metrics can be defined as

$$\begin{array}{c} Acc=\frac{TP+TN}{TP+FP+TN+FN} \end{array}$$ (4)

$$\begin{array}{c} Pre=\frac{TP}{TP+FP} \end{array}$$ (5)

$$\begin{array}{c} Rec\;\left(or\;Sensitivity\right)=\frac{TP}{TP+FN} \end{array}$$ (6)

$$\begin{array}{c} F1=\frac{2}{\frac{1}{Precision}+\frac{1}{Recall}} \end{array}$$ (7)

The accuracy rate provides an overall measure of the number of correct predictions of the model. However, the high accuracy rate of a model does not ensure its ability to distinguish different classes equally if the dataset is imbalanced. In particular, in medical image classification, a model that can be generalized to all classes is required. In such cases, the “precision” and “recall” values provide insight into the performance of the model. “Precision” shows the accuracy of the model’s positive label prediction. This provides the ratio of the correct predictions to the total predictions yielded by the model. Conversely, “recall” measures the percentage of ground truth positives that the model correctly predicted. These two evaluation metrics assess whether the model can reduce the number of FP and FN predictions. “F1-Score” provides a balance between “precision” and “recall,” considering both FPs and FNs. It penalizes extreme values of “precision” and “recall,” each of which is achieved at the expense of the other. Thus, in medical image classification, it is useful to consider evaluation metrics rather than only the accuracy rate to obtain a precise identification of a non-diseased, as well as of a diseased person.

### Implementation

**Fig 7:**
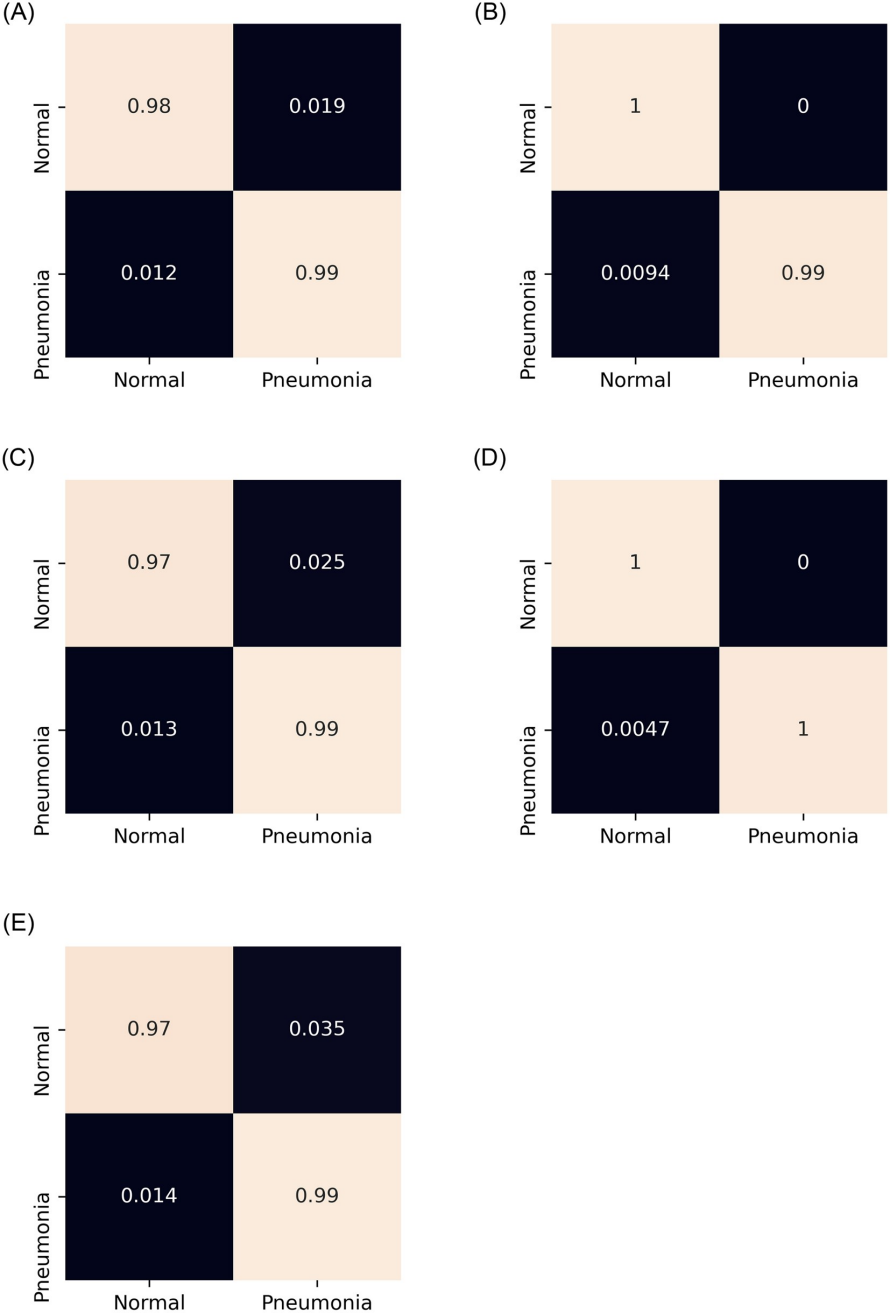
Confusion matrices obtained on the Kermany pneumonia chest X-ray dataset by the proposed method by 5-fold cross validation. a) Fold-1. (b) Fold-2. (c) Fold-3. (d) Fold-4. (e) Fold-5.

**Fig 8:**
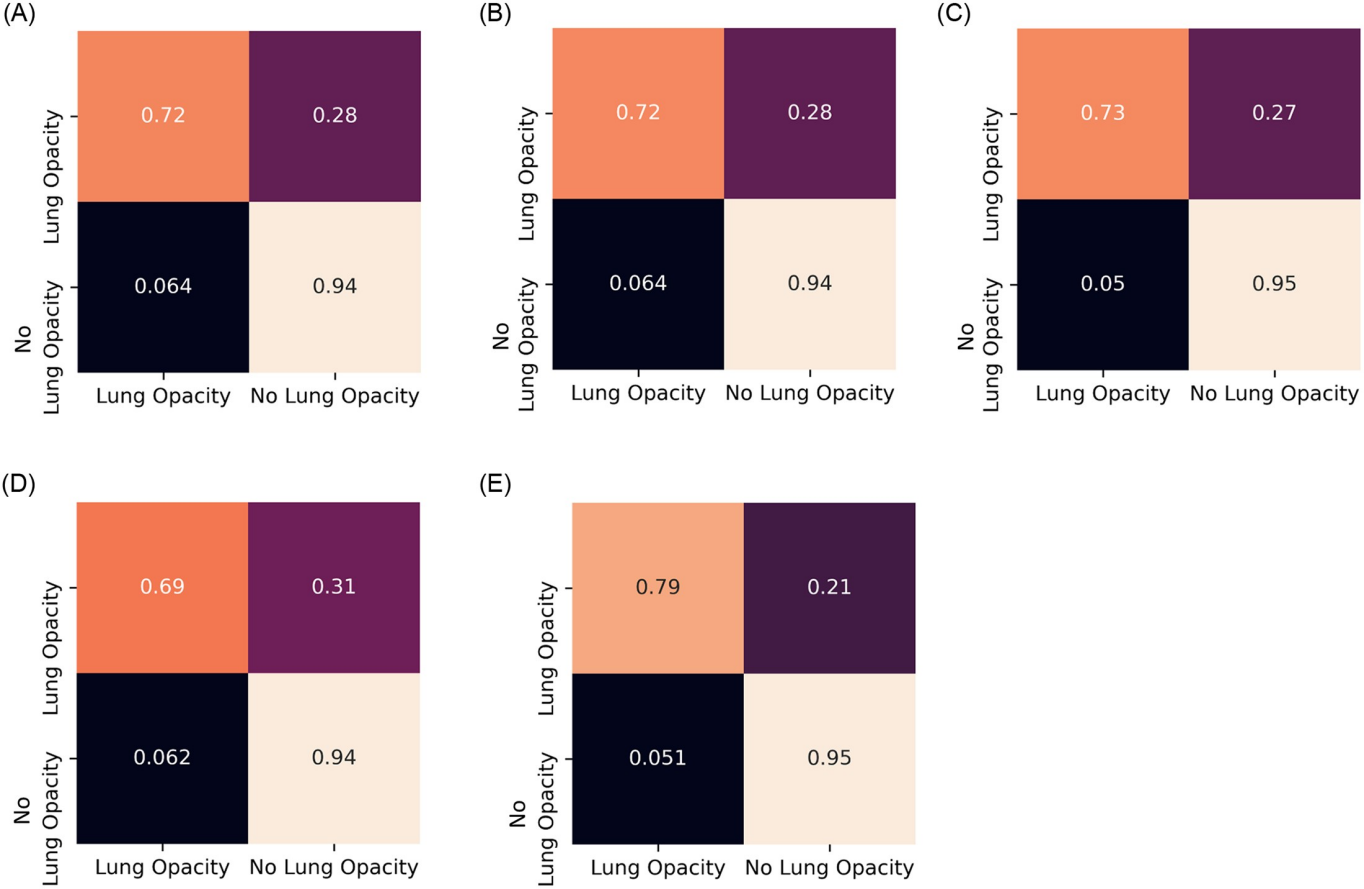
Confusion matrices obtained on the Radiological Society of North America pneumonia challenge chest X-ray dataset by the proposed method by five-fold cross validation. a) Fold-1. (b) Fold-2. (c) Fold-3. (d) Fold-4. (e) Fold-5.

**Fig 9:**
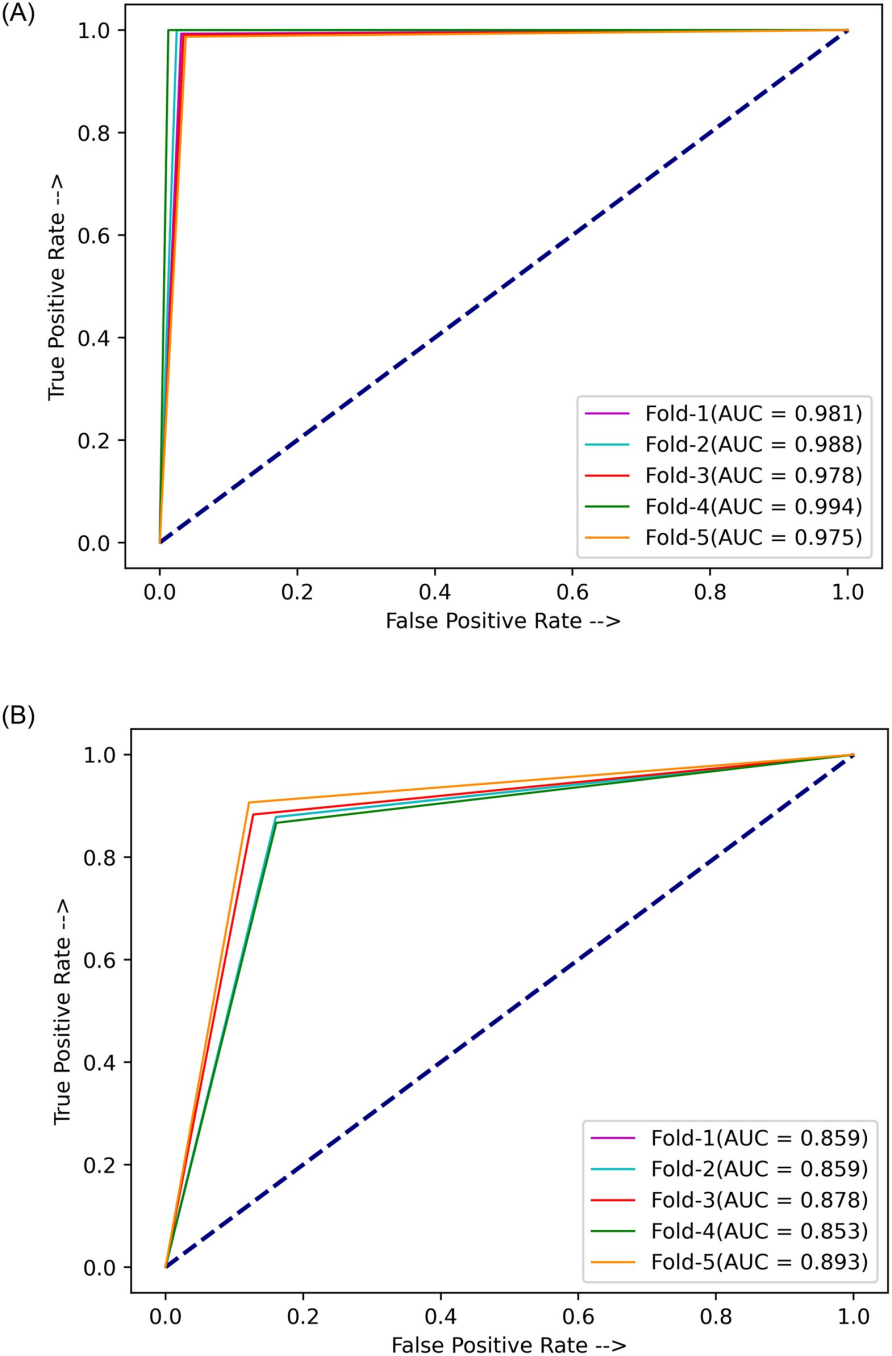
Receiver operating characteristic curves obtained by the proposed ensemble method on the two pneumonia chest X-ray datasets used in this research. (a) Kermany dataset [4]. (b) RSNA challenge dataset [33].

A five-fold cross-validation scheme was used in this study to evaluate robustly the performance of the proposed ensemble model. The results for each fold and the average and standard deviation values over the five folds are tabulated in Table 4 for the Kermany dataset [4] and in Table 5 for the RSNA challenge dataset [33]. The high accuracy and sensitivity (recall) values indicate the reliability of the proposed approach. Further, Figs 7 and 8 show the confusion matrices obtained on the Kermany and RSNA datasets, respectively, and Fig 9 shows the ROC curves obtained by the proposed method on all the five folds of cross-validation on the two datasets.

**Table 4:** Results of five-fold cross-validation of the proposed ensemble method on the pneumonia Kermany dataset [4].

Fold	Acc(%)	Pre(%)	Rec(%)	F1(%)	AUC(%)
1	98.63	98.64	98.63	98.63	98.12
2	99.31	99.33	99.32	99.32	98.82
3	98.38	98.46	98.38	98.29	97.86
4	99.68	99.66	99.66	99.66	99.43
5	98.03	98.03	98.03	98.03	97.54
**Avg±Std. Dev**.	**98.81±0.61**	**98.82±0.59**	**98.80±0.60**	**98.79±0.61**	**98.35±0.68**

*Avg*: average *Std*.*Dev*: Standard Deviation.

**Table 5:** Results of five-fold cross-validation of the proposed ensemble method on the pneumonia Radiological Society of North America challenge dataset.

Fold	Acc(%)	Pre(%)	Rec(%)	F1(%)	AUC (%)
1	86.63	86.78	86.63	86.70	86.63
2	86.78	87.05	87.05	87.05	86.78
3	87.97	88.00	87.80	87.90	87.97
4	85.98	86.00	86.63	86.31	85.98
5	86.89	86.63	86.98	86.80	86.89
**Avg±Std. Dev**.	**86.85±0.72**	**86.89±0.73**	**87.02±0.48**	**86.95±0.59**	**86.85±0.72**

*Avg*: average *Std*.*Dev*: Standard Deviation.

**Fig 10:**
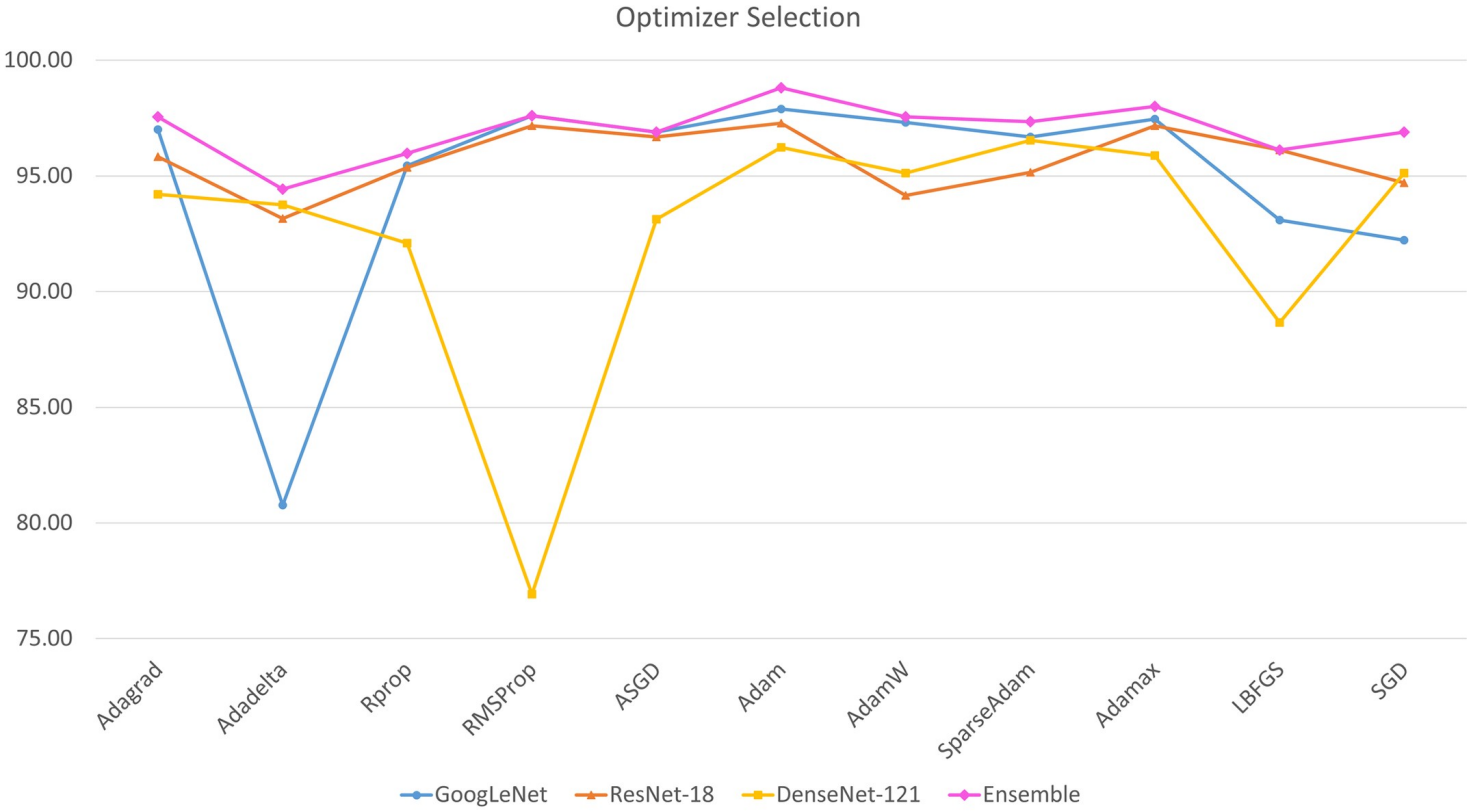
Variation of accuracy rates on the Kermany dataset [4]) achieved by the three base learners, GoogLeNet, ResNet-18, and DenseNet-121 and their ensemble, according to the optimizers chosen for fine tuning.

Fig 10 shows the accuracy rates achieved by the base learners in transfer learning using different optimizers on the Kermany dataset. The best results were obtained by the Adam optimizer for all three base learners; thus, it was chosen as the optimizer to train the base learners for the ensemble framework.

**Fig 11:**
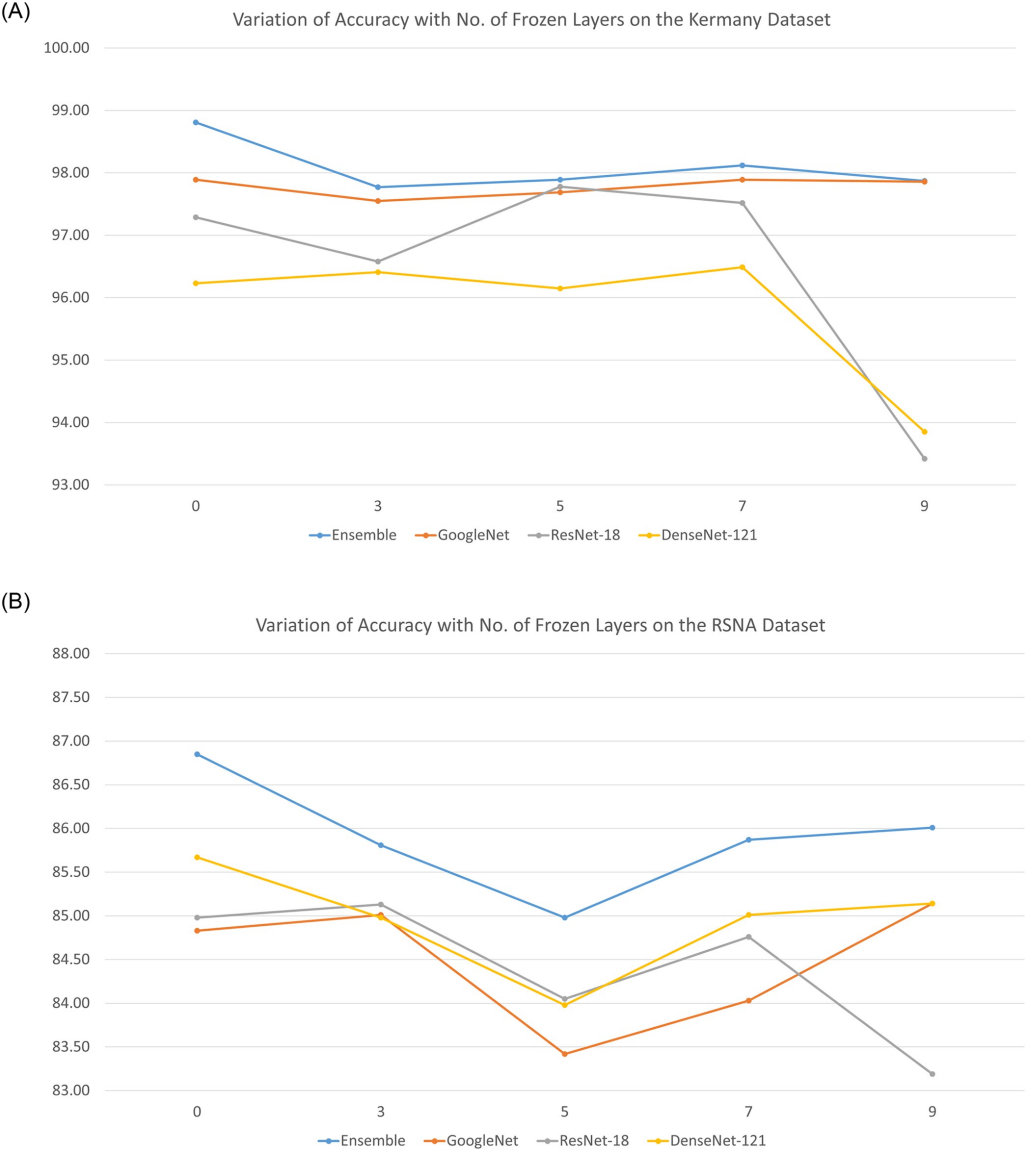
Variation in performance (accuracy rates) of the ensemble with respect to the number of fixed non-trainable layers in the base learners on the two datasets used in this study. (a) Kermany dataset [4]. (b) RSNA challenge dataset [33].

Table 6 shows the results of the various ensembles consisting of three different base learners (including recently proposed architectures), GoogLeNet, ResNet-18, ResNet-50, ResNet-152, DenseNet-121, DenseNet-169, DenseNet-201, MobileNet v2, and NasMobileNet, on the Kermany dataset. The results justify the choice of the combination of base learners used in this study, GoogLeNet, ResNet-18, and DenseNet-121. The ensemble combination achieved an accuracy rate of 98.81%. The next best result, an accuracy rate of 98.54%, was achieved by the ensemble of GoogLeNet, ResNet-18, and MobileNet v2. Further, for the chosen combination of base learners, GoogLeNet, ResNet-18, and DenseNet-121, in the execution of the ensemble we fixed some of the layers and trained the models to select the optimal setting. The results are shown in Fig 11. The best results for the ensemble were achieved when all the layers were trainable (0 layers frozen) on both datasets. Thus, we chose this setting for the ensemble framework.

**Table 6:** Results of extensive experiments performed to determine the base learners for forming the ensemble in this study.

Model-1	Model-2	Model-3	Acc(%)	Pre(%)	Rec(%)	F1(%)
NasNetMobile	MobileNet v2	ResNet-152	96.67	96.70	96.67	96.68
NasNetMobile	MobileNet v2	ResNet-50	97.00	97.02	97.01	97.01
NasNetMobile	MobileNet v2	DenseNet-169	96.41	96.40	96.41	96.41
NasNetMobile	MobileNet v2	DenseNet-201	96.06	96.21	96.07	96.11
MobileNet v2	ResNet-152	DenseNet-169	96.92	97.03	96.92	96.95
MobileNet v2	ResNet-50	DenseNet-169	97.77	97.82	97.78	97.79
MobileNet v2	ResNet-50	DenseNet-201	95.98	96.40	95.98	96.06
MobileNet v2	ResNet-152	DenseNet-201	94.87	95.57	94.87	94.99
NasNetMobile	ResNet-152	DenseNet-169	95.21	95.71	95.21	95.31
NasNetMobile	ResNet-152	DenseNet-201	92.56	94.06	92.56	92.81
NasNetMobile	ResNet-50	DenseNet-169	96.41	96.66	96.41	96.46
NasNetMobile	ResNet-50	DenseNet-201	92.99	94.28	92.99	93.20
GoogLeNet	ResNet-152	DenseNet-121	97.17	97.37	97.18	97.21
GoogLeNet	ResNet-152	DenseNet-201	95.04	95.65	95.04	95.15
GoogLeNet	ResNet-18	DenseNet-201	98.20	98.23	98.21	98.21
GoogLeNet	MobileNet v2	DenseNet-121	98.29	98.29	98.29	98.29
GoogLeNet	ResNet-18	MobileNet v2	98.54	98.54	98.55	98.54
GoogLeNet	MobileNet v2	NasNetMobile	98.12	98.13	98.12	98.11
**GoogLeNet**	**ResNet-18**	**DenseNet-121**	**98.81**	**98.82**	**98.80**	**98.35**

### Gradient-weighted class activation maps analysis

Gradient-weighted class activation maps (GradCAM) [38] were employed in this study to present a visual representation of the distinguishing regions in the chest X-ray images, that is, the regions on which the classifier focuses to make a prediction. CAM calculates the number of weights of each feature map (FM) based on the last convolution layer to compute the contribution of the FM to the prediction $\hat{y}$ at location (*i*, *j*), where our objective is a computed value of $L_{ij}^{g}$ that satisfies $y^{g}=\sum_{i,j}L_{ij}^{g}$. The final FM ($C_{ij}^{k}$) and the prediction $\hat{y}$ are represented through a linear relationship in which the linear layers contain global average pooling (GAP) layers and fully connected layers (FCLs). (1) GAP outputs $A_{k}=C_{ij}^{k}$ and (2) the FCLs, which hold weight $w_{k}^{g}$, generate an output as in Eq 8, where *C*_*k*_ represents the visualization of the *k*^*th*^ FM: $L_{ij}^{g}=\sum_{k}w_{k}^{g}C_{ij}^{k}$.

$$\begin{array}{c} y^{g}=\sum_{k}w_{k}^{g}A_{k}=\sum_{k}w_{k}^{g}\sum_{i,j}C_{ij}^{k}=\sum_{i,j}\sum_{k}w_{k}^{g}C_{ij}^{k} \end{array}$$ (8)

CAM is an unsuitable method because of the problem of the vanishing nonlinearity of classifiers. Thus, instead of pooling them, we use GradCAM for globally averaging the gradients of the FM as weights. While the heat maps are plotted, class-specific weights are collected from the last convolution layer through globally averaged gradients (GAG) of the FM instead of pooling, as in Eq 9, where *P* is the number of pixels in an FM, *g* is the gradient of the class, and $C_{ij}^{k}$ is the value of the *k*^*th*^ FM.

$$\begin{array}{c} \alpha_{k}^{g}=\frac{1}{P}\sum_{i}\sum_{j}\frac{\partial y^{g}}{\partial C_{ij}^{k}} \end{array}$$ (9)

After the relative weights have been gathered, the coarse saliency map (*L*^*c*^) is calculated as the weighted sum, $\alpha_{k}^{c}\times C_{ij}^{k}$, of the ReLU activation (Eq 10), where $\alpha_{k}^{c}$ represents the neuron importance weights. It introduces a linear combination to the FM because only the features that have a positive influence on the respective class are of interest; the negative pixels in the image that belong to other categories are discarded.

$$\begin{array}{c} L^{g}=ReLU(\sum_{i}\alpha_{k}^{g}C^{k}) \end{array}$$ (10)

**Fig 12:**
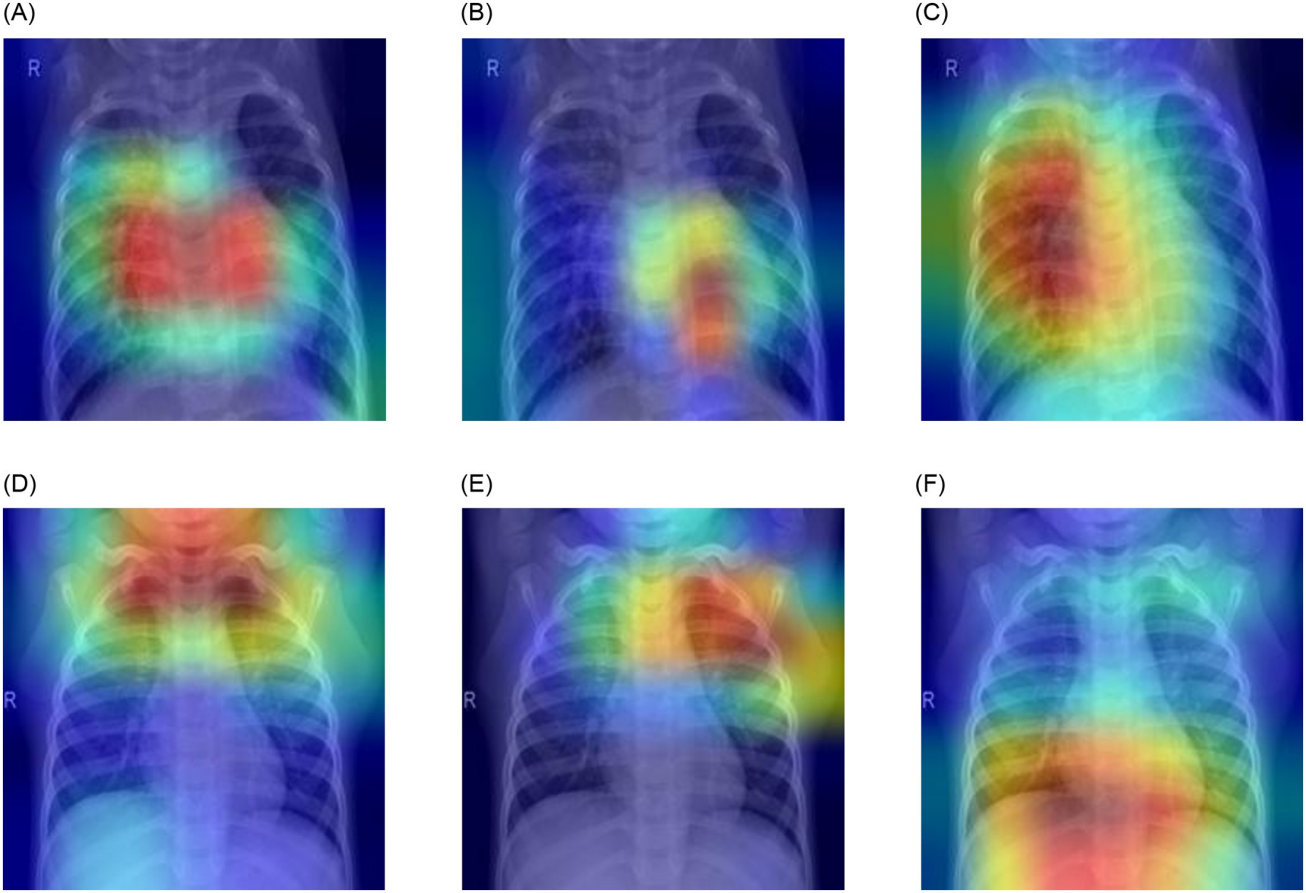
Gradient-weighted class activation map (GradCAM) decision visualization of chest X-ray images when the three chosen base learners were used to form the ensemble. Different regions of the X-rays are the focus of the different models that capture complementary information. Case-1: (a)–(c) show a pneumonic lung X-ray analyzed using the three base learners; the confidence scores of the three base learners are GoogLeNet: 99.99%, ResNet-18: 75.21%, and DenseNet-121: 98.90% Case-2: (d)–(f) show a healthy lung X-ray analyzed using the three base learners; the confidence scores of the three base learners are GoogLeNet: 99.47%, ResNet-18: 97.61%, and DenseNet-121: 98.93%.

Fig 12 shows the results of the GradCAM analysis of a pneumonic and a healthy lung X-ray, where all three models were used to form the ensemble. Evidently, the different models focused on different regions of the lung X-rays, indicating that the base learners capture complementary information. This led to the success of the ensemble approach. The confidence scores for the pneumonic lung X-ray shown in Fig 12(a)–12(c) are GoogLeNet: 99.99%, ResNet-18: 75.21%, and DenseNet-121: 98.90%; all predicted correctly. For the healthy lung case shown in Fig 12(d)–12(f), the confidence scores are GoogLeNet: 99.47%, ResNet-18: 97.61%, and DenseNet-121: 98.93%; all predicted correctly.

### Comparison with state-of-the-art methods

Table 7 compares the performance of the proposed ensemble framework and those of the existing methods in the literature on the Kermany pneumonia dataset. It should be noted that the proposed method outperformed all the other methods. It is also noteworthy that all these previous methods (Mahmud et al. [39], Zubair et al. [13], Stephen et al. [20], Sharma et al. [19], and Liang et al. [11]) revolved around using a single CNN model for the classification of pneumonic lung X-ray images and that the proposed ensemble framework outperformed them, indicating that the ensemble technique devised in this study is a reliable method for the image classification task under consideration. To the best of our knowledge, no studies on the classification of images in the RSNA pneumonia dataset exist. Hence, for this dataset, we compared the performance of the proposed model to that of several baseline CNN models.

**Table 7:** Comparison of the proposed method with other methods in the literature on the Kermany pneumonia dataset [4] and the Radiological Society of North America challenge dataset [33].

Dataset	Method	Acc(%)	Pre(%)	Rec(%)	F1(%)	AUC(%)
Kermany	Mahmud et al. [39]	98.10	98.00	98.50	98.30	-
	Zubair et al. [13]	96.60	97.20	98.10	97.65	-
	Stephen et al. [20]	93.73	-	-	-	-
	Sharma et al. [19]	90.68	-	-	-	-
	Liang et al. [11]	90.50	89.10	96.70	92.70	-
	**Proposed Method**	**98.81**	**98.82**	**98.80**	**98.79**	**98.35**
RSNA	Antin et al. [40]	-	-	-	-	61.00
	Zhou et al. [41]	79.70	-	-	80.00	-
	Yao et al. [42]	-	-	-	-	71.30
	Rajpukar et al. [14]					76.80
	**Proposed Method**	**86.85**	**86.89**	**87.02**	**86.95**	**86.85**

Table 8 shows the evaluation results obtained with the base CNN models used to form the ensemble and several other standard CNN transfer learning models in comparison with those of the proposed method on both the datasets used in this study. It can be seen that the proposed ensemble method outperformed the base learners, as well as other transfer learning models, by a fair margin on both datasets.

**Table 8:** Comparison of the proposed ensemble framework with several standard convolution neural network models in the literature on both the Kermany and the Radiological Society of North America challenge datasets.

Dataset	Model	Acc(%)	Pre(%)	Rec(%)	F1(%)	AUC(%)
**Kermany**	GoogLeNet	97.89	98.12	98.12	98.12	97.89
	AlexNet	97.17	97.22	97.18	97.19	97.17
	VGG-16	97.09	97.12	97.09	97.1	97.09
	DenseNet-121	96.23	96.63	96.24	96.31	96.23
	ResNet-18	97.29	98.31	98.29	98.3	97.29
	**Proposed Method**	**98.81**	**98.82**	**98.80**	**98.79**	**98.35**
**RSNA**	GoogLeNet	84.83	84.98	84.83	84.90	84.83
	AlexNet	85.86	85.15	84.86	85.00	85.86
	VGG-16	81.05	80.08	81.17	80.62	81.05
	DenseNet-121	85.67	84.98	85.55	85.26	85.67
	ResNet-18	84.98	85.55	85.37	85.46	84.98
	**Proposed Method**	**86.85**	**86.89**	**87.02**	**86.95**	**86.85**

Furthermore, to establish the superiority of the proposed ensemble scheme over traditional popular ensemble techniques, the results are compiled in Table 9. The same three base CNN learners, GoogLeNet, ResNet-18, and DenseNet-121, were used in the ensembles; the average results over the five folds of cross-validation are shown for both the Kermany and RSNA challenge datasets. The proposed ensemble method outperformed popular ensemble schemes. On both datasets, it can be seen that the weighted average ensemble that considers only the accuracy metric used as the weights achieved the performance closest to that of the proposed ensemble technique. In the majority voting-based ensemble, the class that obtained the maximum votes from the base learners is predicted as the class of the sample. For the maximum probability ensemble, the probability scores for each class are summed over all the base learners and the class having the maximum probability is set as the predicted class of the sample, whereas in the average probability ensemble, equal weighting is given to each contributing classifier.

**Table 9:** Performance comparison of the proposed ensemble technique and popular ensemble schemes in the literature for the two datasets used. The same base learners were used in all the ensembles: GoogLeNet, ResNet-18, and DenseNet-121.

Dataset	Ensemble technique	Acc(%)	Pre(%)	Rec(%)	F1(%)	AUC (%)
**Kermany**	Maximum Probability	97.77	97.84	97.79	97.76	97.79
	Average Probability	97.85	97.81	97.79	97.78	97.81
	Majority Voting	98.11	98.13	98.12	98.10	97.85
	Weighted Average with only accuracy	98.20	98.22	98.20	98.18	98.11
	**Proposed Ensemble**	**98.81**	**98.82**	**98.80**	**98.79**	**98.35**
**RSNA**	Maximum Probability	85.67	85.80	85.67	85.73	85.67
	Average Probability	86.10	85.98	86.11	86.04	86.11
	Majority Voting	85.98	85.67	85.98	85.82	85.98
	Weighted Average with only accuracy	86.63	86.54	86.63	86.58	86.54
	**Proposed Ensemble**	**86.85**	**86.89**	**87.02**	**86.95**	**86.85**

### Error analysis

**Fig 13:**
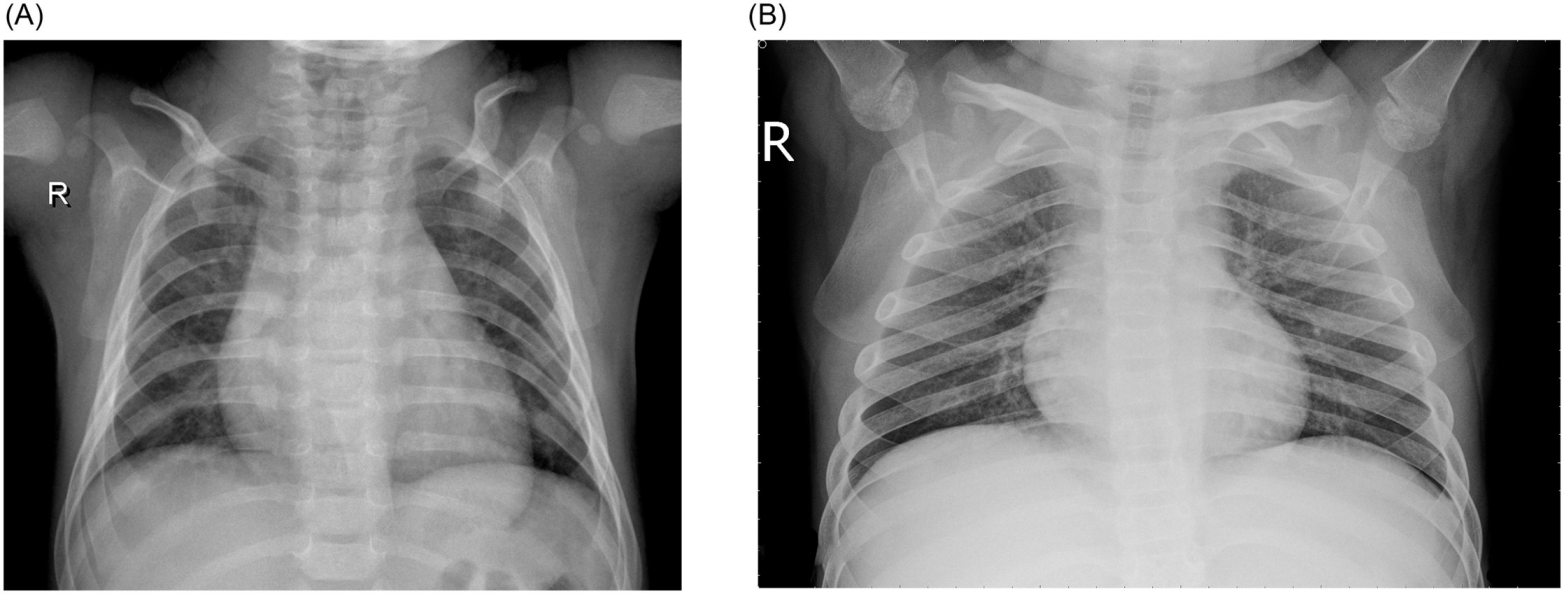
Examples of samples from the Kermany dataset where two out of three base learners yielded incorrect predictions, but the ensemble yielded the correct prediction. Both images are of class “Normal”. **(a) Case-1**: GoogLeNet predicted “*Pneumonia*” with a confidence score of 53.1%, ResNet-18 predicted “*Pneumonia*” with a confidence score of 73.8%, and DenseNet-121 predicted “*Normal*” with a confidence score of 89.4%. The proposed ensemble framework predicted “*Normal*” (correct classification) with a confidence rate of 68.1 **(b) Case-2**: GoogLeNet predicted “*Normal*” with a confidence score of 98.6%, ResNet-18 predicted “*Pneumonia*” with a confidence score of 58.3%, and DenseNet-121 predicted “*Pneumonia*” with a confidence score of 69.3%. The proposed ensemble framework predicted “*Normal*” (correct classification) with a confidence rate of 66.3%.

Fig 13 shows two test samples from the Kermany dataset [4] where two base learners yielded incorrect predictions with a low confidence rate and the third base learner yielded the correct prediction with a very high confidence rate, finally leading the ensemble framework to predict the sample correctly. Fig 13(a) shows a sample where GoogLeNet predicted “Pneumonia” with a confidence score of 52.1%, ResNet-18 predicted “Pneumonia” with a confidence score of 73.8%, and DenseNet-121 predicted “Normal” with a confidence score of 89.4%. The proposed ensemble framework finally correctly predicted the sample to belong to the “Normal” class with a confidence score of 68.1%. Similarly, in the case of Fig 13(b), GoogLeNet predicted “Normal” with a confidence score of 98.6%, ResNet-18 predicted “Pneumonia” with a confidence score of 58.3%, and DenseNet-121 predicted “Pneumonia” with a confidence score of 69.3%. The proposed ensemble framework correctly predicted the sample to be “Normal” with a confidence score of 66.3%. This indicates the robustness of the ensemble framework performance.

**Fig 14:**
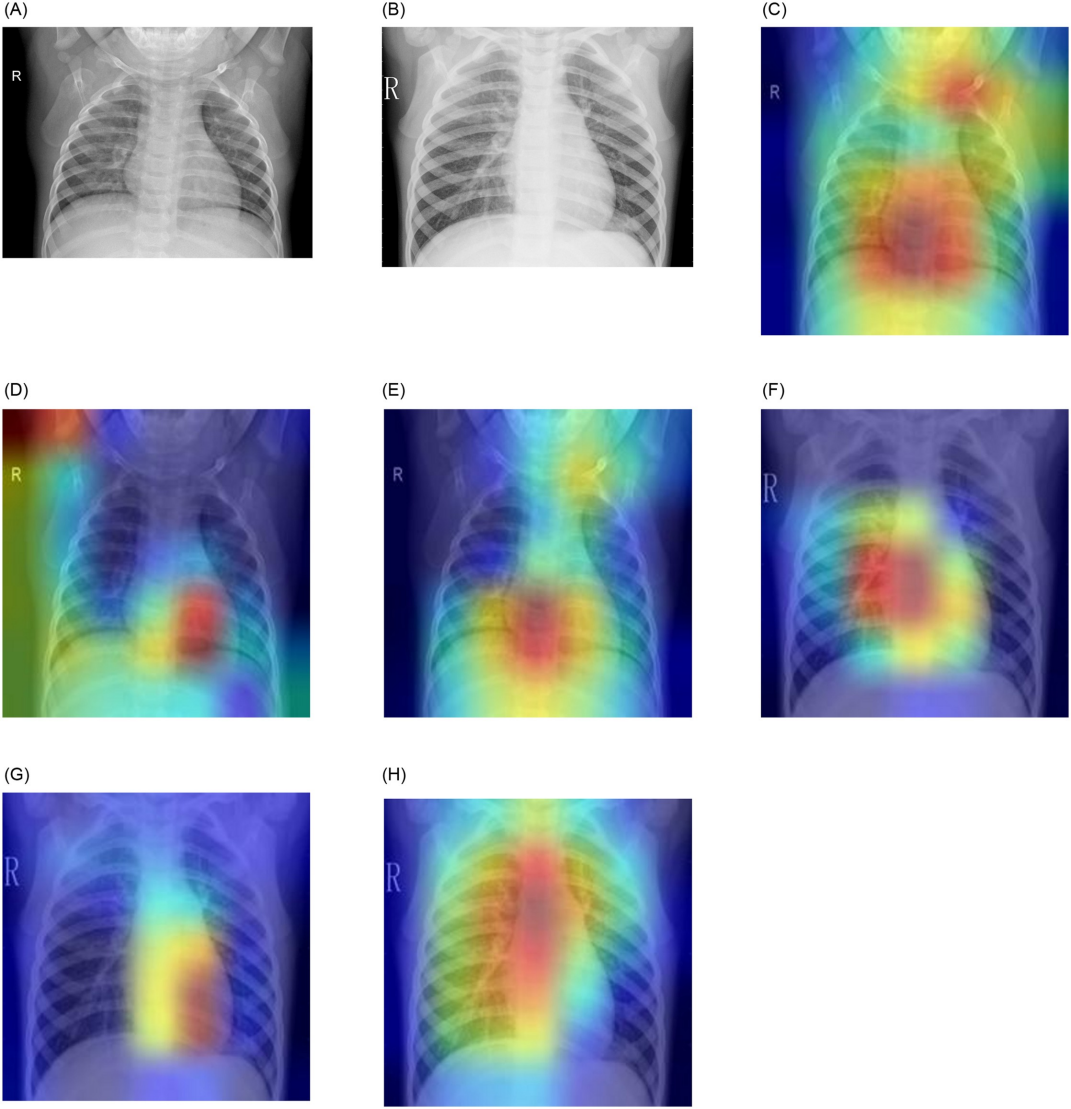
Examples of samples from the Kermany dataset [4] that were classified incorrectly by the proposed ensemble framework. Case-1: (a) shows an image originally belonging to class “Normal” but misclassified as “Pneumonia” by the framework. The GradCAM analysis images are shown in (c), (d), and (e) for GoogLeNet, ResNet-18, and DenseNet-121, respectively. Case-2: (b) shows an image of class “Pneumonia” predicted to belong to the “Normal” class by the framework. The GradCAM analysis images are shown in (f), (g), and (h)for GoogLeNet, ResNet-18, and DenseNet-121, respectively.

Fig 14 shows several test samples from the Kermany dataset [4] where the ensemble framework failed to classify the samples correctly. Fig 14(a) shows a case where a sample belonging to class “Normal” was misclassified as “Pneumonia”; the corresponding GradCAM analysis images are shown in parts (c), (d), and (e). This may be due to the poor image quality, where the contrast of the image is not adequate, resulting in the base learners classifying the sample incorrectly. The GradCAM analysis showed that GoogLeNet and DenseNet-121 focused on the spinal cord in the X-ray, whereas ResNet-18 focused on the white area of the retracted lungs, leading to incorrect predictions. Fig 14(b) shows a case where an image of class “Pneumonia” was classified as “Normal” by the model. The GradCAM analysis images are shown in (f), (g), and (h). As in the previous case, the GoogLeNet and DenseNet-121 models focused on the spinal cord, and the ResNet-18 model focused on part of the spinal cord and the retracted left lung. A pneumonic lung X-ray is characterized by abscesses or pleural effusion, that is, fluid in the alveoli, which appears as white spots in a lung X-ray, as explained in Fig 1. It is plausible that such an early stage of pneumonia, where the white infiltrates have just started to appear sparingly in the lungs, was not captured by the CNN models. In such cases, doctors use air bronchogram signs to detect pneumonia. The shape and lumen of the bronchi with air bronchogram signs were used to distinguish lung cancer, tuberculosis, and pneumonia.

### Statistical analysis

To analyze statistically the viability of the proposed ensemble framework, we performed two non-parametric tests: McNemar’s statistical test [43] and the analysis of variance (ANOVA) test [44], where the proposed ensemble model was compared to the base classifiers, the probability scores of which were used in this study to determine the formation of the ensemble: GoogLeNet, ResNet-18, and DenseNet-121. Table 10 tabulates the McNemar’s test results and Table 11 tabulates the ANOVA test results on both the pneumonia chest X-ray datasets used in this study. To reject the null hypothesis, the p-value in both McNemar’s and the ANOVA test should be lower than 0.05 (5%); according to Tables 10 and 11, for every case in both datasets, the *p*−*value* is less than 0.05. Thus, the null hypothesis was rejected by the results of both statistical tests. This establishes that the proposed ensemble framework captures complementary information from the base classifiers and its predictions are superior, thus ensuring that the ensemble model is statistically dissimilar to any of the contributing models.

**Table 10:** Results of McNemar’s statistical test of the ensemble model and the base learners on both datasets. For all the base learners with which the proposed model is compared, the *p*−*value* is less than 0.05, and thus, the null hypothesis is rejected.

McNemar’s Test	p-value	
	Kermany dataset	RSNA dataset
**GoogLeNet**	0.0000	0.0017
**ResNet-18**	0.0430	0.0214
**DenseNet-121**	0.0002	0.0006

**Table 11:** Results of analysis of variance (ANOVA) statistical test of the ensemble model and the base learners on both datasets. For all the base learners with which the proposed model is compared, the *p*−*value* is less than 0.05, and thus, the null hypothesis is rejected.

ANOVA Test	p-value	
	Kermany Dataset	RSNA Dataset
**GoogLeNet**	0.0435	0.0131
**ResNet-18**	0.0021	0.0001
**DenseNet-121**	0.0017	0.0056

## Conclusion and future work

Early detection of pneumonia is crucial for determining the appropriate treatment of the disease and preventing it from threatening the patient’s life. Chest radiographs are the most widely used tool for diagnosing pneumonia; however, they are subject to inter-class variability and the diagnosis depends on the clinicians’ expertise in detecting early pneumonia traces. To assist medical practitioners, an automated CAD system was developed in this study, which uses deep transfer learning-based classification to classify chest X-ray images into two classes “Pneumonia” and “Normal.” An ensemble framework was developed that considers the decision scores obtained from three CNN models, GoogLeNet, ResNet-18, and DenseNet-121, to form a weighted average ensemble. The weights assigned to the classifiers were calculated using a novel strategy wherein four evaluation metrics, precision, recall, f1-score, and AUC, were fused using the hyperbolic tangent function. The framework, evaluated on two publicly available pneumonia chest X-ray datasets, obtained an accuracy rate of 98.81%, a sensitivity rate of 98.80%, a precision rate of 98.82%, and an f1-score of 98.79% on the Kermany dataset and an accuracy rate of 86.86%, a sensitivity rate of 87.02%, a precision rate of 86.89%, and an f1-score of 86.95% on the RSNA challenge dataset, using a five-fold cross-validation scheme. It outperformed state-of-the-art methods on these two datasets. Statistical analyses of the proposed model using McNemar’s and ANOVA tests indicate the viability of the approach. Furthermore, the proposed ensemble model is domain-independent and thus can be applied to a large variety of computer vision tasks.

However, as previously mentioned, in some instances the ensemble framework failed to produce correct predictions. In the future, we may investigate techniques such as contrast enhancement of the images or other pre-processing steps to improve the image quality. We may also consider using segmentation of the lung image before classification to enable the CNN models to achieve improved feature extraction. Furthermore, because three CNN models are required to train the proposed ensemble, the computation cost is higher than that of the CNN baselines developed in studies in the literature. In the future, we may attempt to reduce the computational requirements by employing methods such as snapshot ensembling.

## Abbreviations

*Acc*: Accuracy
*AI*: Artificial Intelligence
AUC: Area Under the Curve
CAD: Computer-Aided Diagnosis
CNN: Convolutional Neural Network
*F*1: F1-Score
FCL: Fully Connected Layer
FM: Feature Map
GAG: Globally Averaged Gradients
GAP: Global Average Pooling
Grad-CAM: Gradient-weighted Class Activation Maps
*Pre*: Precision
*Rec*: Recall
ReLU: Rectified Linear Unit
ROC: Receiver Operating Characteristics
RSNA: Radiological Society of North America
